# Neurological Presentation of Giant Pituitary Tumour Apoplexy: Case Report and Literature Review of a Rare but Life-Threatening Condition

**DOI:** 10.3390/jcm11061581

**Published:** 2022-03-13

**Authors:** Valentina Puglisi, Elisabetta Morini, Fiammetta Biasini, Luisa Vinciguerra, Giuseppe Lanza, Placido Bramanti

**Affiliations:** 1 Department of Neurology and Stroke Unit, Istituti Ospitalieri, ASST Cremona, Viale Concordia 1, 26100 Cremona, Italy; valentina.puglisi@asst-cremona.it (V.P.); luisa.vinciguerra@asst-cremona.it (L.V.); 2 IRCCS Centro Neurolesi “Bonino-Pulejo”, Via Provinciale Palermo, Contrada Casazza, 98124 Messina, Italy; elisabetta.morini@irccsme.it (E.M.); placido.bramanti@irccsme.it (P.B.); 3 Unit of Neurology and Neuromuscular Diseases, Policlinico University Hospital “G. Martino”, Via Consolare Valeria 1-2, 98124 Messina, Italy; fiammetta.biasini@gmail.com; 4 Department of Surgery and Medical-Surgical Specialties, University of Catania, Via Santa Sofia 78, 95123 Catania, Italy; 5 Clinical Neurophysiology Research Unit, Oasi Research Institute-IRCCS, Via Conte Ruggero 73, 94018 Troina, Italy

**Keywords:** giant pituitary adenoma, pituitary apoplexy, acute hypopituitarism, neuroimaging

## Abstract

Background: Giant pituitary adenomas are benign intracranial tumours with a diameter ≥4 cm. Even if hormonally non-functional, they may still cause local extension, leading to symptoms that include mostly gland dysfunction, mass effects, and, much less frequently, apoplexy due to haemorrhage or infarction. Neurological presentation of giant pituitary tumour apoplexy is even more rare and has not been systematically reviewed. Case Presentation: An 81-year-old woman was admitted to the Emergency Department because of acute onset headache, bilateral visual deficit, and altered consciousness. Computed tomography showed a giant mass lesion (>5.5 cm diameter) expanding upward to the suprasellar cistern, optic chiasm, and third ventricle, over-running the sphenoid sinus, and with lateral invasion of the cavernous sinus. Laboratory investigations revealed central adrenal and hypothyroidism insufficiency, while magnetic resonance imaging confirmed a voluminous suprasellar tumour (~6 cm diameter), with signs of pituitary tumour apoplexy. Neurological manifestations and gland-related deficits improved after hormonal replacement therapy with a high dose of intravenous hydrocortisone, followed by oral hydrocortisone and levo-thyroxine. The patient declined surgical treatment and follow-up visit. Conclusions: Giant pituitary tumour apoplexy is a rare but potentially life-threatening condition. Prompt diagnosis and multidisciplinary management may allow a remarkable clinical improvement, as seen in this case.

## 1. Introduction

Giant pituitary adenomas are a subset of benign tumours that present mainly as non-functioning pituitary adenomas (NFPAs) and account for approximately 6–10% of all pituitary tumours [1]. NFPAs usually arise from adenopituitary cells and do not cause hormonal hypersecretory syndrome or other gland-related dysfunctions [2]. Although usually benign and relatively rare, giant NFPAs can be problematic due to their large size, invasiveness, common extrasellar extension, and other factors that make them difficult to manage. Moreover, technical difficulties and procedural issues that may arise during surgical resection can expose patients to a high risk of complications.

The clinical spectrum of giant pituitary adenomas varies widely, ranging from totally asymptomatic presentation (and consequently often diagnosed late) to severe hypothalamic and/or pituitary dysfunction and neurological symptoms due to the mass effect. Mechanical compression of the remaining anterior pituitary gland or of the pituitary stem can also result in the gradual development of partial or complete hypopituitarism. Apart from the endocrinological effects, the neurological manifestations secondary to compression of adjacent structures include headache, visual field defects, ophthalmoplegias, and much less frequently, pituitary apoplexy (PA) [3,4]. 

PA is thus a rare but life-threatening condition caused by acute infarction or haemorrhage of a pituitary adenoma. NFPAs appear to be at higher risk of apoplexy, with an incidence of 0.2–0.6 events/100 persons per year [5]. It is worth noting that although very rare, apoplexy represents the first clinical manifestation in 80% of cases of previously unknown pituitary adenomas [6] and, among NFPAs, 8% of cases presented with clinical evidence of PA [7]. Indeed, the rapid enlargement of pituitary tumours causes gradual and progressive compression of the adjacent parasellar structures and typically manifests with acute-onset headache, visual defects, cranial nerve palsy, changes in consciousness, and endocrine dysfunctions [8]. As such, tumour-related PA is a serious complication requiring prompt identification and appropriate management.

The incidence of PA may vary across different types of pituitary adenoma. For instance, Nielsen et al. retrospectively reviewed 192 consecutive patients with a large suprasellar, clinically inactive, operated adenoma [9]. Based on the neurological symptoms and findings at operation, a diagnosis of PA was found to be considerably more frequent than previously reported. In contrast, a recent single-centre case series of 33 patients with PA as the first manifestation of both functioning adenomas and NFPAs [10] confirmed this condition was rare. The apparent discrepancy may be explained by the heterogeneity of the study cohorts, which ranged from PAs that occurred in micro- or macroadenomas to those occurring in functioning adenomas or NFPAs. Further studies are therefore needed to clarify the actual incidence of PA.

Here, we describe the case of a giant NFPA complicated by PA. In this context, there are very few literature reports of giant NFPAs presenting solely with PA. A PubMed-based literature review found that only five case reports [11,12,13,14,15] and a case series of four adult patients [16] had been published from 1990 to November 2021. The other reports of PA were in functioning pituitary macroadenomas and were therefore excluded; also excluded were cases reporting PA in pituitary adenomas where the tumour size was not specified. Even more rare and not systematically described in the literature is the neurological presentation of a giant pituitary tumour apoplexy. In the present study, we report such a case and review the literature in order to draw attention to this rare but challenging condition. 

## 2. Case Presentation

An 81-year-old woman was admitted to the Emergency Department (ED) because of acute-onset headache, altered consciousness, and hematuria. At admission, she appeared lethargic and confused, and her arterial blood pressure was 150/90 mmHg. Neurological examination did not show any sign of meningeal irritation or ophthalmological dysfunction, except for a bilateral vision loss. Apart from the above-mentioned manifestations, the clinical examination was otherwise normal. The patient also suffered from bilateral glaucoma, arterial hypertension, and atrial fibrillation, and was treated with antihypertensive (ramipril) and anticoagulant (acenocoumarol) drugs, respectively. Family and psychosocial history were negative. 

**Figure 1:**
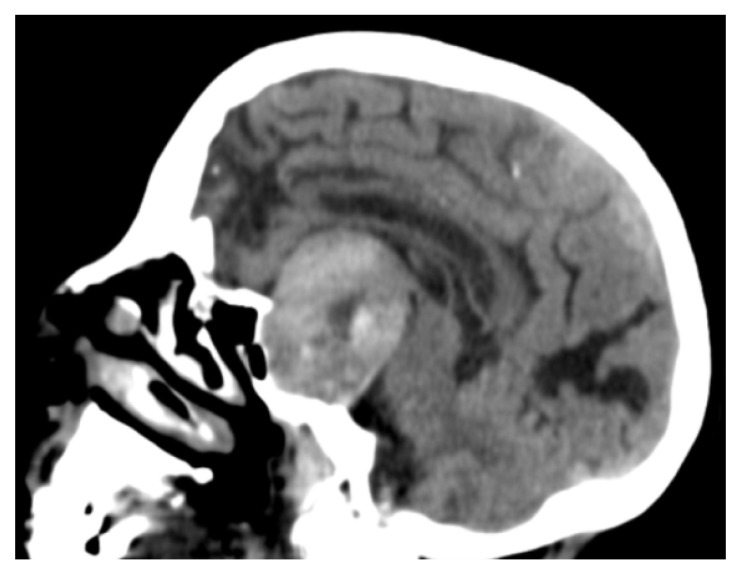
Sagittal brain computed tomography scan showing a giant mass lesion (>5.5 cm diameter) expanding upward to the suprasellar cistern and to the third ventricle, over-running the sphenoid sinus, and with lateral invasion of the cavernous sinus.

**Figure 2:**
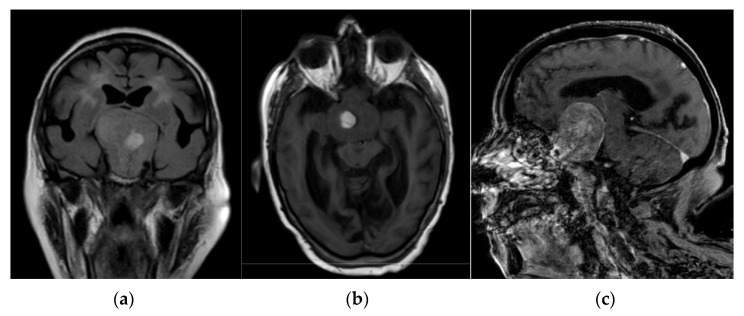
Brain magnetic resonance imaging showing a voluminous suprasellar tumour (Grade III according to Goel et al. [15]) with a mixed pattern of solid and liquid components (colliquative and adipose degeneration areas). This suggests the presence of a tumour PA due to haemorrhage within a pre-existing pituitary adenoma: (**a**) coronal Fluid-attenuated Inversion Recovery; (**b**) axial T1-weighted; (**c**) sagittal T1-weighted with Gadolinium.

Routine laboratory test results, including electrolytes and glucose, were normal except for an INR of 8.51, which was compatible with an uncontrolled anticoagulation therapy. An urgent computed tomography (CT) scan of the brain showed a giant mass lesion (>5.5 cm diameter) expanding upward to the suprasellar cistern and the third ventricle, over-running the sphenoid sinus, and with lateral invasion of the cavernous sinus (Figure 1). Magnetic resonance imaging (MRI) confirmed a voluminous suprasellar tumour (Grade III, according to Goel et al. [17]), near the optic chiasm, with a mixed pattern of solid and liquid components (colliquative and adipose degeneration areas), suggesting the presence of PA due to haemorrhage within a pre-existing adenoma (Figure 2).

The conclusion from urgent endocrine evaluation was adrenal and thyroid insufficiency, as shown by a cortisol level of 137 mmol/l (normal range: 138–690), ACTH 3 pg/mL (normal range: 5–27), GH < 0.1 ng/mL (normal range: 0.13–9.88), TSH 0.09 µU/mL (normal range: 0.4–4.0), fT3 0.56 pmol/l (normal range: 2.7–6.4), and fT4 6.47 pmol/l (normal range: 8–20). The level of prolactin was normal at 5 ng/mL (normal range: 3–27). A suspicion of prolactinoma due to the so-called “hook effect” was ruled out by carrying out a dilution test with a serum prolactin sample. Prompt administration of hormonal replacement therapy, comprising a high dose of intravenous (IV) hydrocortisone (administered before the tests results were available) followed by oral hydrocortisone and levo-thyroxine, resulted in remarkable improvement of the neurological and endocrinological manifestations. The patient became fully alert and her confused state and headache both resolved. Because the patient declined surgical treatment, conservative management based on medical therapy and clinical observation was adopted. A short-term clinical, laboratory, and instrumental follow-up was scheduled, but she did not attend the visit.

## 3. Discussion

We report here the case of a patient with a rare giant pituitary adenoma (~6 cm diameter) who experienced the even more rare complication of a PA-associated neurological presentation, likely due to an uncontrolled anticoagulation therapy-related haemorrhage. NFPAs are pituitary adenomas without hormonal hypersecretion that may be micro-, macro- or giant adenomas. However, giant pituitary adenomas that present exclusively with PA are considered very rare [18]. Among the different subtypes of pituitary tumours, the occurrence of apoplexy tends to favour non-functioning adenomas [19,20]. Functioning adenomas are usually detected promptly due to several signs of hormonal secretion before the occurrence of bleeding or infarction, whereas NFPAs are typically diagnosed at a later stage. As a consequence, they can continue to progressively grow and reach a considerable size before diagnostic detection [21]. The NFPA reported here fits into the latter scenario. In some cases, the extent of necrosis due to the apoplectic event may be so large that it destroys the hormone-producing tumour cells, thus masking a previously hormone-producing adenoma. However, the patient described here did not show any clinical sign that was suggestive of a functioning adenoma.

Although PA mostly occurs spontaneously, precipitating factors can be identified in 10–40% of cases [19]. These include sudden changes in blood flow within the pituitary gland (e.g., angiography, myelography, lumbar puncture, spinal anaesthesia, systemic hypertension, pituitary irradiation, closed-head trauma, orthopaedic or cardiac surgery), an imbalance between stimulation of the pituitary gland and increased blood flow within the pituitary adenoma (e.g., dynamic pituitary testing, pregnancy), and anticoagulated states (e.g., coagulopathies, anticoagulant drug intake including vitamin K antagonist or new oral anticoagulants, platelet inhibitors, and thrombolytic agents) [22,23]. Of these precipitating factors, arterial hypertension [24,25] and uncontrolled anticoagulant therapy [6,26] are the most common, as in the case described here. Review of the literature revealed other precipitating factors in adult patients, such as surgery in two cases described by Goel et al. [12] and in four cases reported by Ahmad et al. [16], endocrine stimulation tests in one case [9], and post-partum in one case [13]. Fanous et al. and Romano et al. published two cases of spontaneous PA [14,15]. 

It should be highlighted that PA as an exclusively presenting symptom is very uncommon in the literature published to date (reviewed in Table 1). Indeed, the presence of a giant pituitary adenoma was not known prior to the clinical manifestation of PA in the cases reported by Perotti et al., Fanous et al., and Romano et al. [13,14,15]. In contrast, PA occurred in a previously known pituitary tumour in the other published case reports and case series [11,12,16].

As described in different case series of typical PAs, the most common features at presentation are sudden onset headache and ophthalmoplegia, followed by hypopituitarism [24,25,27]. In the case reported here, the patient also showed altered consciousness and hematuria, likely due to the mass effect and uncontrolled anticoagulation therapy, respectively. Although the neurological examination did not show any specific optic dysfunction, a visual defect due to compression of the optic chiasm may have been present. It is indeed likely that such a large sellar mass with suprasellar extension could have affected the visual field. Accordingly, large tumours are often identified due to visual impairment or other mass effect symptoms [28]. However, the bilateral vision loss due to a known glaucoma in the patient’s past medical history might have led to a bias in this evaluation. In this context, it should also be highlighted that the clinical presentation of PAs in elderly patients differs from that seen in younger patients. For example, visual disturbances in the elderly can be confused with other age-related disorders, such as cataracts, macular degeneration, retinal vascular diseases, and glaucoma, as occurred in our patient. Similarly, symptoms of hypogonadism in elderly subjects with prolactinomas are often overlooked and only large tumours are diagnosed because of mass effect symptoms. Moreover, hypopituitarism in the elderly may be misdiagnosed because of overlapping age-related symptoms or because it misleads the interpretation of hormonal values [28].

PA occurs mostly in a previously unknown history of pituitary mass. Making a prompt diagnosis can therefore be challenging given its similarities with many neurological disorders and other life-threatening conditions, thus often leading to late identification and delayed management. The two most important differential diagnoses are subarachnoid haemorrhage secondary to aneurysm rupture, and bacterial meningitis. Other conditions to exclude are suprasellar aneurysm, intracerebral haemorrhage, hypertensive encephalopathy [29], and cavernous sinus thrombosis [22,30]. Typically, the symptoms of mechanical compression and endocrine dysfunction evolve a few hours to 48 h after onset of the apoplexy, although a subacute course is also possible [31]. 

Based on these considerations, the assessment of endocrine function through basal and dynamic tests in patients with pituitary adenoma is mandatory in order to identify those requiring replacement therapies or other specific treatments [32,33]. The co-morbid conditions present in the case reported here, particularly the atrial fibrillation not controlled by proper anticoagulation therapy, could have misled the diagnosis despite the typical clinical presentation of headache, altered consciousness, and hormonal dysfunction. Neuroimaging is therefore crucial for early detection, differential diagnosis and prompt management; CT and MRI are helpful for the identification of NFPA and PA.

PA can be managed by surgery or in select cases by conservative treatment, but a multidisciplinary team should always be involved in any decision-making process. Conservative management requires immediate IV administration of high-dose glucocorticoids, even before the tests results are available. This is especially important for hemodynamically unstable cases or for patients with altered consciousness or severe neurological impairment. In the present case, hormone replacement therapy significantly improved both the neurological (i.e., headache and altered consciousness) and endocrinological manifestations (i.e., adrenal and thyroid insufficiency). This approach covers not only the increased risk of hypoadrenalism, but also exerts anti-inflammatory and anti-oedematous effects. Since corticotropic deficiency is present in the vast majority of patients at the onset of PA and may be life threatening even if treated surgically or conservatively, IV corticosteroids should be given as soon as the diagnosis is confirmed [6,34,35,36]. 

Regarding the replacement therapy with GH, there is hardly any information on the treatment with recombinant human growth hormone (rhGH) of old patients with GH deficiency, as was the woman reported here. As previously systematically reviewed [37], two studies only included patients older than 80 years, but their data could not be extracted. Moreover, the effects of rhGH substitution on conventional cardiovascular risk factors are still controversial and studies on the effects of rhGH in elderly patients with GH deficiency on clinically relevant endpoints, e.g., cardiovascular morbidity, fractures and mortality, have not been reported. As such, to date, there is no convincing evidence on the safety and efficacy of rhGH treatment in this age group and, therefore, we did not consider the possibility to replace GH in this case.

Surgical treatment of pituitary adenomas usually includes both microscopic and endoscopic trans-sphenoid approaches. However, because of their extension and involvement of neurovascular structures, these tumours often pose significant surgical challenges and are associated with increased treatment morbidity and mortality, increased recurrence rates, and overall poor outcome and long-term prognosis [36,38]. Careful postoperative surveillance of patients following partial removal of a pituitary adenoma is therefore crucial. Accordingly, in a follow-up study of 32 patients with NFPAs presenting with typical acute PA, the post-surgery recurrence rate in those not receiving postoperative radiotherapy was 11.1% at just over 5 years [27]. 

Finally, the course of PA is highly variable. When the diagnosis is delayed, and therefore also decompression and corticosteroid treatment, death may occur as a result of adrenal failure or neurological complications. As such, PA should be considered as a medical emergency and any patient with a suspicion of hypoadrenalism needs to start treatment with hydrocortisone [19]. Prompt therapy with steroid and levo-thyroxine improves the level of consciousness and the symptoms of hypopituitarism, as shown in the patient described here. Following discharge, the patient reported clinical stability and a follow-up visit was scheduled but was not attended by the patient. Thus, we acknowledge the lack of follow-up for a large tumour with apoplexy and managed conservatively limits this report. Similarly, no surgery was performed and, therefore, histopathology is not available.

In this context, the literature review points towards a favourable long-term follow-up in the two cases reported by Perotti et al. [11] and Fanous et al. [12], with a near complete-to-complete clinical resolution of post-partum PA and spontaneous PA treated with steroids and radical surgery, respectively. Conversely, Goel et al. [10] and Ahmad et al. [14] described a series of cases who underwent re-surgery of a known residual pituitary tumour complicated by PA, with unfavourable outcomes (exitus). This variability in the patients’ outcome highlights the need for a prompt medical therapy and, when possible, a radical tumour resection.

## 4. Conclusions

Giant tumour PA is a rare condition that can present with neurological signs of mass effects and corticotropic deficiency. The case reported here can be viewed as a model for a rare but life-threatening disorder that requires early diagnosis and prompt management, together with close multidisciplinary collaboration.

## Figures and Tables

**Table 1:** Summary of previously published articles on giant non-functioning tumour pituitary apoplexy (PA).

Study, Year	Age, Years	Sex	Known Tumour	Precipitating Factors for PA	Clinical Presentation	Neuroimaging Findings	Management and Outcome
Okuda, et al., 1994 [9]	60	Female	Yes	Endocrine stimulation tests	Headache, stuporous status, hemiparesis	Haemorrhage intra- and extra-tumour	Surgery + radiation, with partial tumour residual; follow-up not available
Goel, et al., 1995 [10]	40	Male	Yes	Surgery of pituitary tumour	Coma	Diffuse swelling and haemorrhage	Re-surgery, followed by exitus (3 days after)
	17	Male	Yes	Surgery of pituitary tumour	Visual deficit, coma	Diffuse swelling and haemorrhage	Re-surgery, followed by exitus (3 months after)
Ahmad, et al., 2005 [14]	50	Male	Yes	Surgery of pituitary tumour	Altered mental status	Diffuse swelling and haemorrhage	Re-surgery, followed by exitus (3–20 days after)
	30	Male	Yes		Altered mental status		
	35	Female	Yes		Third nerve paresis, visual deficit		
	19	Male	Yes		Altered mental status		
Perotti, et al., 2010 [11]	29	Female	No	Post-partum	Headache, vomiting, coma	Haemorrhage intra-tumour	Steroids + surgery; functionally independent at 6-month follow-up
Fanous, et al., 2013 [12]	39	Male	No	Spontaneous	Headache, diplopia, cranial nerve palsy	Necrotic apoplexy	Steroids + surgery, near complete resolution at 2-month follow-up
Romano, et al., 2020 [13]	65	Male	No	Spontaneous	Visual deficit, altered mental status, hemiparesis	Tumour apoplexy	Endoscopic approach; follow-up not available

## Data Availability

Data are contained within the article.
